# Glycosylated Foot‐And‐Mouth Disease Virus‐Like Particles Produced in *Pichia Pastoris* Enhance Stability and Immunogenicity

**DOI:** 10.1111/1751-7915.70271

**Published:** 2025-11-24

**Authors:** Zhiyao Li, Hu Dong, Shuanghui Yin, Manyuan Bai, Zhidong Teng, Lingbo Chen, Suyu Mu, Yun Zhang, Yaozhong Ding, Shiqi Sun, Huichen Guo

**Affiliations:** ^1^ State Key Laboratory for Animal Disease Control and Prevention College of Veterinary Medicine, Lanzhou University, Lanzhou Veterinary Research Institute, Chinese Academy of Agricultural Sciences Lanzhou China; ^2^ Gansu Province Research Center for Basic Disciplines of Pathogen Biology Lanzhou China

**Keywords:** assembly, foot‐and‐mouth disease, glycosylation, *Pichia Pastoris*, virus‐like particles

## Abstract

Despite the availability of vaccines, foot‐and‐mouth disease (FMD) remains a significant concern in many developing countries, causing severe economic losses and affecting local farming communities. Virus‐like particle (VLP) vaccines are highly regarded for their safety and efficacy. N‐glycosylation for stabilisation and recognition by antigen‐presenting cells has been a widely adopted strategy, particularly in enveloped viruses. Here, FMD virus (FMDV) VLPs were employed as a model for artificial glycosylation. N‐glycosylation was introduced by mutating the potential glycosylation site of VP1 and then N‐glycosylated FMDV VLPs were successfully produced in *Pichia pastoris*. Glycan profiling revealed that the majority of associated glycans (72.93%) were of the high‐mannose type, with additional hybrid type (4.16%) and complex type (22.92%) detected. Functional analyses demonstrated that glycosylation significantly enhanced the stability of VLPs and facilitated the uptake by antigen‐presenting cells. Animal experiments further revealed that glycosylation could induce a higher cellular immune response compared to WT VLPs, offering a reference for the glycosylation design of VLP vaccines.

## Graphical abstract

An N‐glycosylation was strategically introduced at the 166th position of VP1 via artificial mutagenesis. The glycans mainly consist of high‐mannose type, significantly enhancing the stability and uptake of VLPs. The glycosylated VLPs conferred a robust Th1‐type immune response in mice and pigs and provided 100% protection against FMDV in pigs.

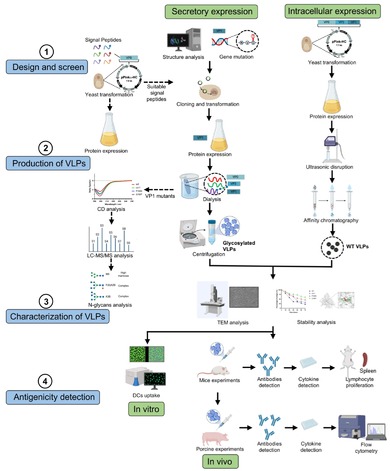

## 1 Introduction

Foot‐and‐mouth disease (FMD) is an acute, highly contagious disease caused by the FMD virus (FMDV). Belonging to the *Picornaviridae* family and the *Aphthovirus* genus, FMDV is a nonenveloped virus that exhibits an icosahedral structure with a diameter of 25–30 nm and a sedimentation coefficient of 146S. Additionally, several intermediates are formed during replication, such as 75S empty capsids, 12S pentamers and 5S protomers (Dong et al. 2021). Currently, the predominant types of FMDV in China are O and A (Zhu, Zou, et al. 2018). Outbreaks of FMD have resulted in the reduction of cloven‐hoofed animal production capacity and significant economic losses in affected regions. Inactivated vaccines have played a crucial role in the prevention and control of FMD (Uddowla et al. 2012). However, their production entails risks of virus leakage. Virus‐like particles (VLPs), which are morphologically similar to natural virions but lack viral genetic material, offer a biosafe alternative (Mignaqui et al. 2019). Thus, developing VLPs and refining their physicochemical properties and immunogenicity have been increasingly emphasized.

Natural glycosylation modifies over 50% of proteins, categorised into four major types: N‐linked, O‐linked, C‐linked, and glycosylphosphatidylinositol (Ohtsubo and Marth 2006; Li et al. 2015). Notably, 75% of glycoproteins are N‐glycosylated in eukaryotes. Among them, N‐glycosylation exhibits a distinctive amino acid motif, Asn‐Xaa‐Ser/Thr (Xaa represents any amino acid except Pro) (Kasturi et al. 1995). Protein stability is markedly enhanced when a phenylalanine residue precedes the glycosylated asparagine in reverse turns, forming motifs such as FNXS/T, FXNXS/T, or FXXNXS/T (Culyba et al. 2011). Eighty‐five percent of the glycoproteins expressed in *P. pastoris* are Man_8‐14_GlcNAc_2_ (Imperiali and O'Connor 1999), making this yeast an indispensable eukaryotic platform for heterologous protein expression and glycosylation engineering (Srivastava et al. 2023).

N‐glycosylation is crucial for the physicochemical properties, stability and recognition of proteins. The N‐glycosylation of xylanase at position 124 is essential for pH tolerance and thermal stability (Chang et al. 2017). Additionally, glycosylation levels significantly impact the substrate affinity of purple acid phosphatase (Desko et al. 2009). Dendritic cells (DCs) and other myeloid cells possess C‐type lectin receptors (CLRs), including DC‐SIGN, DEC‐205, langerin, and dectin‐1, which recognize polysaccharides to facilitate antigen uptake (Taylor and Drickamer 2003; Plato et al. 2013). This receptor‐mediated endocytosis by antigen‐presenting cells (APCs) enhances antigen processing and presentation to lymphocytes, highlighting how polysaccharide modification can potentiate immunogenicity via improved APC uptake.

Glycoproteins have played a significant role in virus recognition, assembly and escape, especially in enveloped viruses (Hyakumura et al. 2015; Casas‐Sanchez et al. 2021). Notably, even the naturally unglycosylated proteins or capsids of nonenveloped viruses can be engineered for glycosylation to improve their biophysical properties and immunogenicity. This strategy has been applied to noroviruses (Hanisch 2022), hepatitis E viruses (Graff et al. 2008), rotaviruses (Caust et al. 1987; Estes and Cohen 1989), enterovirus 71 (Zhao et al. 2017) and human papillomavirus (Zhou et al. 1993). Glycosylated VLPs, produced in *P. pastoris* via the expression of the 112–608 aa of ORF2 of the nonenveloped virus Hepatitis E Virus (HEV), elicited favorable immune responses in mice (Gupta et al. 2020). Given these precedents and the fact that FMDV, as a nonenveloped virus, presents an ideal model for glycosylation engineering, investigating whether mannose glycosylation can augment FMDV VLP immunogenicity holds significant biotechnological promise.

Our study investigated the potential for enhancing the immunogenicity of FMDV VLPs through artificial glycosylation. We show that the introduced amino acid changes do not affect the secondary structure of VP1 and the mutated VP1 can be assembled into VLPs. Glycans are displayed in the external space of VLPs, and in vitro experiments demonstrated that glycosylation promoted the uptake of glycosylated VLPs by APCs. Although the glycosylated VLPs showed no significant difference in specific and neutralising antibody titers compared with nonglycosylated VLPs in mice and pigs, except that G166T VLPs exhibited improved neutralisation in mice, they elicited more robust Th1‐type cellular immune responses. Notably, immunisation with glycosylated VLPs provided 100% protection in pigs. This study offers a novel approach for the preparation of glycosylated vaccines and the glycosylation design of nonenveloped viruses.

## 2 Experimental Procedures

### 2.1 Cells, Plasmids and Strains

The pPink‐P1 (containing N1017D + H2145Y), pPink‐HC and other secretory plasmids were preserved at the Lanzhou Veterinary Research Institute. The secretory plasmids, whose signal peptides are derived from *Saccharomyces cerevisiae* α‐mating factor, *Pichia pastoris* β‐glucanase, *Saccharomyces cerevisiae* flocculin, *Saccharomyces cerevisiae* acid phosphatase, *Saccharomyces cerevisiae* glucanosyltransferase and *Wickerhamomyces ciferrii* α‐mating factor were named pPink‐αHC, pPink‐DSE4, pPink‐FLO10 pPink‐PHO5 pPink‐GAS and pPink‐SP27 respectively. The PichiaPink strain1 (ade2) was purchased from Invitrogen (Grand Island, NY, USA). The pSMKVP0, pSMAVP1 and pSMCVP3 plasmids used for expressing *E. coli‐*derived VLPs are preserved in our lab (Guo et al. 2013). Immortalised DC2.4 cell line (SCC142, murine bone marrow‐derived) was purchased from Merck (Rahway, United States) and grown as previously described (Lu et al. 2024). The FMDV strain (O/BY/CHA/2010) was propagated in BHK‐21 cells as previously described (Xie et al. 2019).

### 2.2 Plasmid Construction and Yeast Transformation

**FIGURE 1:**
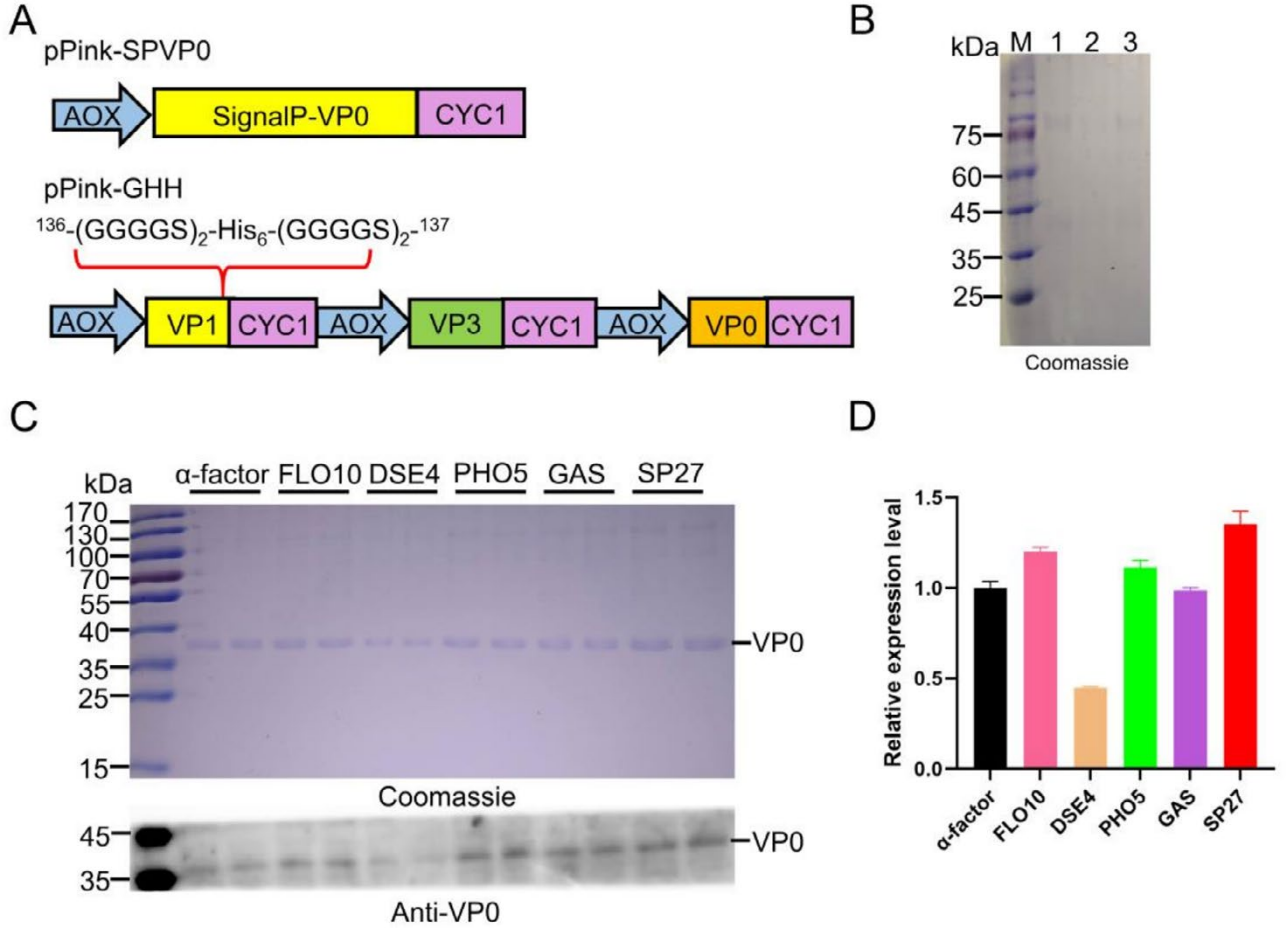
Schematic of the pPink‐SPVP0, pPink‐GHH and screening of signal peptides for VP0. (A) Schematic representation of the plasmids employed in this study. (B) SDS‐PAGE analysis of background proteins in untransformed yeast. Lane M: Marker, Lane 1–3: Untransformed strains. (C) SDS‐PAGE and WB analysis of the expression levels of VP0 mediated by different signal peptides. The pPink‐GHH was constructed with a His_6_ inserted between the 136th and 137th of VP1, and (GGGGS)_2_ linker sequences added on both ends of His_6_. (D) Relative expression level analysis of VP0. The relative secretion levels were quantified using ImageJ. Data are presented as mean ± standard deviation of 3 biological replicates.

The VP0, VP3 and VP1 genes were amplified from pPink‐P1 and inserted into pPink‐HC by *Eco*RI/*Kpn*I. The VP1 and VP3 expression cassettes were engineered into the pPink‐VP0 plasmid using *Bgl*II/*Bam*HI. Then, the His_6_ comprising (GGGGS)_2_ at its N and C termini was inserted into VP1 at the 136th, yielding plasmid pPink‐GHH (Figure 1A). The VP0 gene was cloned into plasmids containing different signal peptides, resulting in plasmids pPink‐SPVP0. Similarly, the VP3 and VP1 genes containing L51T, P160N and G166T amino acid changes were engineered respectively into pPink‐SP27, generating plasmids pPink‐SPVP3 and pPink‐SPVP1. The constructed plasmids were linearized with *Spe*I and then transferred to PichiaPink strain1 by electroporation following the manufacturer's guidelines.

### 2.3 Purification of FMDV VLPs

Wild type (WT) VLPs were intracellularly expressed in yeast strain using the pPink‐GHH (Li et al. 2025). Following ultrasonic disruption, the supernatant was centrifuged at 15,800 × *g* (Eppendorf 5810R centrifuge, equipped with F‐34‐6‐38 fixed‐angle rotor for 50 mL tubes) for 30 min. The sample was then subjected to dialysis and affinity chromatography. After ultrafiltration with phosphate‐buffered saline (PBS), the VLPs were quantified by ELISA (Harmsen et al. 2005). In brief, 96‐well plates were coated overnight with 0.5 μg trapping antibody M170 (Dong et al. 2022) diluted with carbonate buffer (pH 9.6) at 4°C. Serially diluted antigen samples were then added to the plates and incubated at 37°C for 1 h, followed by the guinea pig anti‐FMDV antibodies and the horseradish peroxidase (HRP)‐conjugated rabbit anti‐guinea pig IgG and incubated at 37°C for 30 min. Finally, the OD_450nm_ value was measured. According to the standard curve generated based on the known FMDV virion concentration, the concentration of VLPs was then calculated. Assembly efficiency was measured as the ratio of VLP amount to purified capsid protein amount.

To generate glycosylated VLPs, the supernatant of secreted VP0, VP3, or the glycosylated VP1 was centrifuged at 12,000 × *g* for 30 min, respectively, and filtered through 0.22 μm membranes to remove cellular debris. Then, the supernatant was concentrated using 10 kDa ultrafiltration tubes (Millipore, Billerica, USA). After that, the concentrated proteins were mixed in an assembly buffer (300 mM NaCl, 10 mM Tris, 50 mM KCl, 2 mM MgCl_2_, 1% Triton X‐100, 0.1 mM PMSF 0.1 mM DTT, pH 8.0) at a ratio of 1:1:1 for 48 h, followed by ultrafiltration with PBS.

### 2.4 Transmission Electron Microscopy

Ten microliters of VLP samples were added to 200‐mesh copper grids and adsorbed at room temperature for 1 min. Next, they were negatively stained with 3% phosphotungstic acid for 1 min. The sample was observed with a Hitachi H‐7100FA (Tokyo, Japan) transmission electron microscope (TEM) (operating at 80 kV with a field emission gun).

### 2.5 Analysis of N‐Glycans Using LC–MS/MS

N‐glycans analysis was performed using an Ultimate 3000 (Thermo Fisher) with an analytical column ACQUITY UPLC Glycan BEH Amide (130 Å, 1.7 μm, 2.1 × 150 mm). Mobile phase A was 50 mM ammonium formate (pH 4.4), and mobile phase B was acetonitrile (ACN). The flow rate was 0.5 mL/min. Samples were analysed using a Q Exactive Hybrid Quadrupole Orbitrap Mass Spectrometer (Thermo Fisher). MS1 resolution was 70,000, the acquisition range was 200–2000 m/z, the AGC target was 1 × 10^6^, and the maximum injection time was 50 ms. MS2 resolution was 17,500, and the AGC target was 1 × 10^5^. Samples were processed by LC–MS/MS and data were collected using Xcalibur. Raw files were subjected to analysis using the Peptide Mapping Analysis module of Biopharminder software.

### 2.6 Cellular Uptake

DCs were seeded in 24‐well plates at a volume of 500 μL (1 × 10^6^ cells per well) and incubated at 37°C until approximately 80% confluency was reached. Subsequently, the cells were incubated with 1 μg of WT VLPs, L51T VLPs, P160N VLPs and G166T VLPs, respectively, for 1 h, 2 h and 6 h. After incubation, the cells were analysed for indirect immunofluorescence assay (IFA) as follows: the cells were washed three times with sterile PBS, fixed with 4% paraformaldehyde for 15 min, and permeabilized with 0.1% Triton X‐100 solution at room temperature for 15 min. The cells were then washed three times with PBST (5 min per wash), blocked with 5% newborn bovine serum (NBS) for 1 h, and sequentially incubated with pig‐derived FMD positive serum diluted in 1% NBS (1:100) and FITC‐conjugated goat anti‐pig antibody (1:200) at 37°C for 1 h. Finally, the cells were stained with DAPI at room temperature for 15 min. The uptake of VLPs by DCs was evaluated based on the observed FITC fluorescence intensity using an inverted fluorescence microscope.

### 2.7 Animal Experiments

Fifty‐four 6‐week‐old female BALB/c mice were randomly divided into 6 groups of 9 mice each (5 mice for serum antibody detection, 1 mouse as a backup and 3 mice for lymphocyte proliferation assay). Then, ten micrograms of WT VLPs, L51T VLPs, P160N VLPs, and G166T VLPs were emulsified with ISA 201 adjuvant and intramuscularly injected into mice with a single dose of 200 μL. PBS served as a negative control (PBS), and ten micrograms of *E. coli* ‐derived VLPs (endotoxin content < 50 EU/mL, prepared in our laboratory) were injected as a positive control (PC).

Nineteen two‐month‐old FMDV‐negative pigs were kept in a biosafety level 3 (BSL‐3) laboratory for 3 days before being randomly divided into 4 groups, each group housed in a separate barn. PBS group: sterile PBS (2 mL), PC group: *E. coli* ‐derived VLPs (50 μg), WT group: yeast‐derived nonglycosylated VLPs (50 μg), Gly group: glycosylated G166T VLPs (50 μg). All pigs were intramuscularly injected with a single dose of 2 mL (VLP groups emulsified with ISA 201) at the ear‐root‐neck area and bled at 7, 14, 21 and 28 dpi. After 28 days of immunisation, all pigs were intramuscularly challenged with 2 mL of strain O/BY/CHA/2010 (1000 ID_50_) at the ear‐root‐neck area and observed for 7 days post‐challenged.

### 2.8 Detection of Specific Antibodies by ELISA

The specific antibodies were determined by sandwich ELISA. Rabbit anti‐FMDV antibody was diluted 1:1000 with 50 mM carbonate buffer (pH 9.6), coated overnight at 4°C, after being washed, treated with 1% bovine serum albumin (BSA), and incubated at 37°C for 1 h. The plates were then incubated with 100 μL inactivated O FMDV (3 μg/mL) at 37°C for 1 h. Serum samples at a dilution of 1:8 were added and incubated at 37°C for 1 h. After being washed, they were incubated with HRP‐conjugated rabbit anti‐mouse IgG (for mouse samples) or HRP‐conjugated rabbit anti‐pig IgG (for pig samples) at 37°C for 30 min. 3,3′,5,5′‐tetramethylbenzidine (TMB) was then added and reacted in the dark for 15 min followed by 2 M H_2_SO_4_ to terminate, and then OD_450nm_ was measured by spectrophotometer (Biotek, Vermont, USA). The antibody titers were Log_10_‐transformed.

### 2.9 Microplate Neutralisation Assay

The neutralising antibodies were determined by microplate neutralisation assay. Briefly, the serum samples were inactivated at 56°C for 30 min. The serially diluted serum was mixed with 100 TCID_50_ O/BY/CHA/2010 and incubated at 37°C for 1 h. The mixture was then added to BHK‐21 cells in 96‐well plates and incubated for 72 h. After incubation, the lesions in each well were observed by the inverted microscope, and the neutralising antibody titers were calculated using the Reed–Muench method.

### 2.10 Lymphocyte Proliferation Assay

The T‐lymphocyte proliferation assay was performed with the Cell Titre 96AQueous Non‐Radioactive Cell Proliferation Assay (Promega, Madison, WI, USA). In brief, mouse spleens were aseptically isolated at 28 dpi and lymphocyte suspensions were prepared with 2 × 10^6^ cells in 1 mL and added to 96‐well plates at a concentration of 100 μL per well. Furthermore, phytohemagglutinin (PHA) at a final concentration of 10 mg/L was used as a positive control, while the untreated cells were used as a negative control, and the RPMI 1640 medium as a blank. Additionally, one microgram of VLPs was added to the test group. Each group had three replicates. The 96‐well plates were incubated at 37°C for 44 h; after incubation, [3‐(4,5‐dimethylthiazol‐2‐yl)‐5‐(3‐carboxymethoxyp‐henyl)‐2‐(4‐sulfophenyl)‐2H‐tetrazolium, inner salt (MTS)] (working concentration: 5 mg/mL) was added at 10 μL per well for 4 h. The absorbance at 490 nm was measured using a spectrophotometer. The lymphocyte stimulation index (SI) was calculated as the ratio of the mean value of the test group to that of the negative control group.

### 2.11 Flow Cytometry

Porcine peripheral blood mononuclear cells (PBMCs) were differentiated at 28 dpi as previously described with modifications (McCullough et al. 1999). In brief, PBMCs from each pig were isolated from whole blood using a PBMC isolation kit for pigs (Solarbio, Beijing, China) and seeded into 96‐well U‐plates (2 × 10^6^ cells per well), stimulated using VLPs. Subsequently, the live cells were labelled using Fixable Reactive Dye eFluor 780 (Thermo Fisher), and then incubated with mAb against CD3 (BD Pharmingen), CD4 (BD Pharmingen) and CD8ɑ (Southern Biotech) at 4°C for 30 min. Next, the differentiation of lymphocytes was detected by flow cytometry (Mu et al. 2024).

### 2.12 Cytokine Detection

The levels of IFN‐γ in mouse serum were determined using a commercially available mouse IFN‐γ kit (R&D Systems, Minnesota, USA). Similarly, the levels of IFN‐γ, TNF‐α, IL‐4 and IL‐1β in porcine serum were determined using a commercial ELISA kit (Lianke Bio, Hangzhou, China) by referring to the instructions.

### 2.13 Statistics

The data presented in this paper were analyzed by the one‐way analysis of variance (ANOVA) using GraphPad Prism 8 (Dotmatics, Boston, USA). Here, *p* < 0.05 was considered statistically significant.

## 3 Results

### 3.1 Screening of Signal Peptides and Potential Glycosylation Sites

To determine whether VP0, VP3, and VP1 contain putative signal peptides, SignalP 5.0 (DTU, Lyngby, Denmark) was used for prediction. Our analyses indicated that there was no typical signal peptide sequence in any of them (data not shown). N‐glycosylation sites were subsequently predicted using NetNGlyc 1.0 (DTU, Lyngby, Denmark), which revealed that the glycosylated sequon N‐X‐T/S existed at positions 13, 132 and 238 of VP0. Notably, VP3 lacked identifiable glycosylation sequons, whereas VP1 harboured a potential site at position 100. Using Discovery Studio software (BIOVIA, San Diego, USA), we performed the in silico screening of VP1 for N‐, T‐ and S‐residues (Tables S1–S3), generating 16 potential candidates for amino acid changes ranked by post‐mutation free energy changes. To further assess the probability of these sites, PyMOL (Schrödinger, New York, USA) was utilized. Apart from position 51 (C‐strand) and 166 (H‐strand), glycosylation sites formed by amino acid changes at positions 160 are located in the disordered regions of VP1, and all three sites are exposed on the surface of VLPs (Figure S1).

Previous studies have shown that VP0 exhibits higher expression efficiency in *P. pastoris* than VP3 or VP1, prompting us to utilise VP0 as a model protein for signal peptide screening (Li et al. 2025). Different plasmids were constructed: pPink‐SPV0 expresses secretion‐competent VP0 proteins with the SP27 signal peptide, while pPink‐GHH enables intracellular co‐expression of VP1, VP3 and VP0 (Figure 1A). To detect the background proteins of yeast cells, the untransformed *Pichia* cells were used as a negative control (Figure 1B). Following the induction of VP0 vectors with varying signal peptides, a comparison was made with the secretion capacity provided by the signal sequence derived from the α‐factor. The results revealed that the secretion capacity of DSE4 was approximately 30% of that of the α‐factor. The other four signal peptides exhibited a slight improvement relative to α‐factor, and SP27 exhibited a significant enhancement, achieving 1.3 ‐fold higher secretion capacity than the α‐factor control (Figure 1C,D).

### 3.2 Expression of Structural Proteins of FMDV in *P. Pastoris*

**FIGURE 2:**
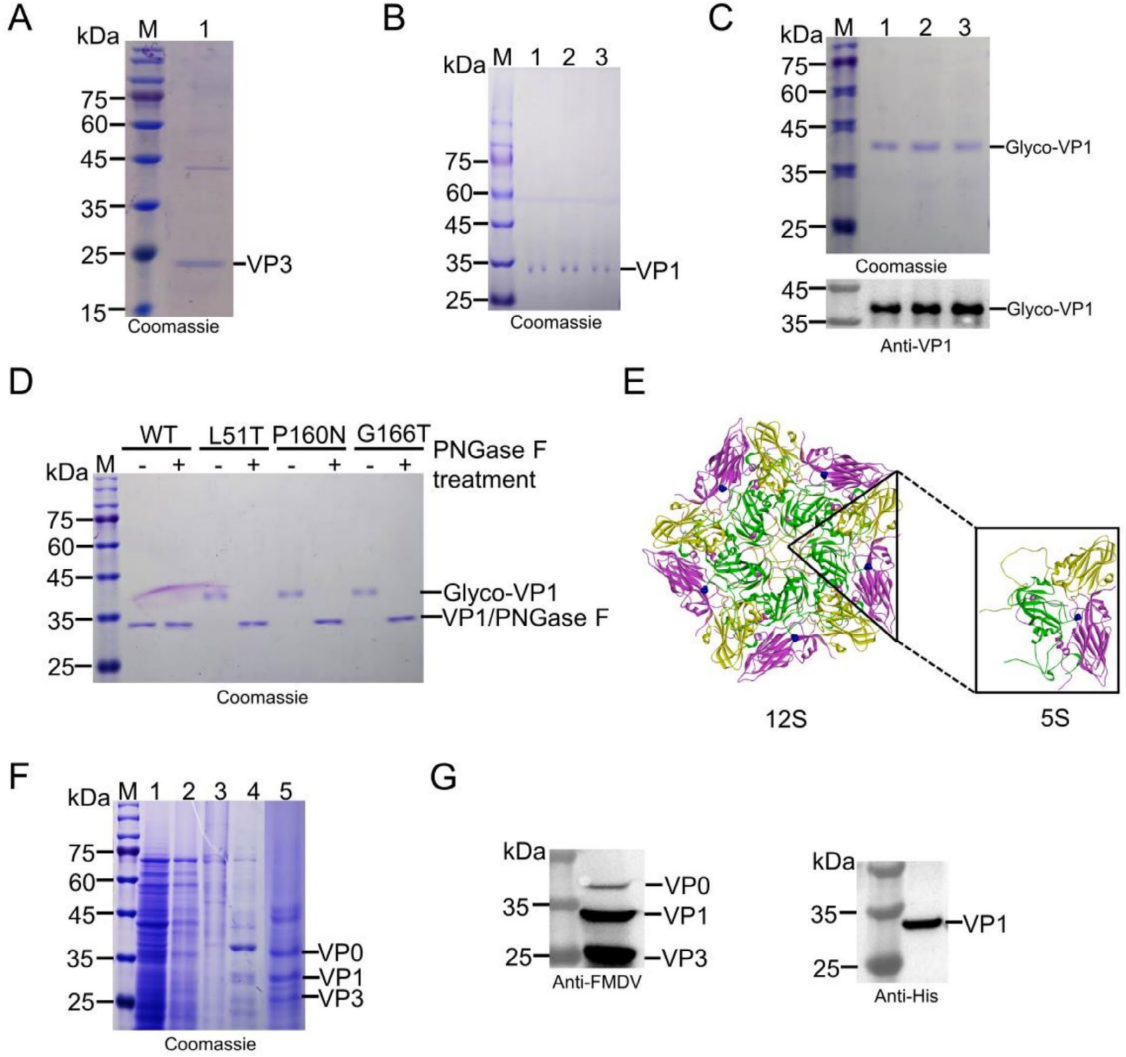
Secretory and intracellular expression analysis of FMDV structural proteins in *P. pastoris*. SDS‐PAGE analysis of VP3 (A) and VP1 (B) secretory expression. (C) SDS‐PAGE and WB analysis of glycosylated VP1. Glyco‐VP1: Glycosylated VP1. (D) SDS‐PAGE analysis of glycosylated VP1 treated with PNGase F. (E) The position of His_6_ in the 5S and 12S after being inserted at residue 136 of VP1. The images were generated using PyMOL, PDB: 7enp. The insert position is blue, VP0 is magenta, VP1 is green, VP3 is yellow. (F) Purification and characterisation of VLPs derived from pPink‐GHH. Lane M: Marker, Lane 1: Supernatant after disruption, Lane 2: Flow‐through fraction, Lane 3: 20 mM imidazole eluent, Lane 4: 300 mM imidazole eluent, and Lane 5: Ultrafiltration concentrates. (G) WB analysis of VLPs derived from pPink‐GHH.

To determine whether SP27 is also capable of mediating the secretion of VP3 or VP1, we constructed VP3 and VP1 secretion expression plasmids containing SP27 and VP1 mutant plasmids containing L51T, P160N, and G166T amino acid changes. After induction, the medium was analysed by SDS‐PAGE. The results revealed the presence of bands at approximately 25 and 35 kDa, consistent with the secretion of VP3 and VP1, respectively (Figure 2A,B). To verify whether glycosylation could be formed upon amino acid changes in VP1, the mutant strains were expressed and then analysed by SDS‐PAGE. The results indicated the presence of a band near 38 kDa. Western blot (WB) analysis using anti‐VP1 antibodies confirmed that this migrated band was VP1 (Figure 2C). The glycosylated proteins were treated with PNGase F followed by SDS‐PAGE revealing restoration of the native protein size (Figure 2D). The results demonstrated that SP27 could effectively secrete VP3 and VP1. The authentic glycosylation site at position 100 of VP1 was not recognised, while VP1 proteins with the introduced potential glycosylation sites migrated at a higher molecular weight, suggesting that the introduced sites were glycosylated.

For comparison, nonglycosylated VLPs were also prepared. pPink‐GHH was electrotransformed into yeast and induced. The purified proteins were detected by WB using anti‐FMDV serum as the primary antibody, then three bands were observed near 25–37 kDa. However, only one band was found near 35 kDa after detection using an anti‐His_6_ antibody; as expected, only VP1 is His6‐tagged (Figure 2E–G), suggesting that structural proteins were successfully expressed from pPink‐GHH. Notably, the three structural proteins VP0, VP1 and VP3 can be co‐purified via VP1 His6‐tag mediated affinity chromatography, suggesting that the three proteins assemble into VLPs.

### 3.3 Assembly and Characterisation of WT VLPs and Glycosylated VLPs

VP1 and its mutants were analyzed using circular dichroism (CD) to determine whether the secondary structure of VP1 is affected by the mutation. The results showed that the secondary structures were similar, indicating that these amino acid changes and N‐glycan had a minimal impact on the secondary structure of VP1 (Figure S2 and Table S4).

**FIGURE 3:**
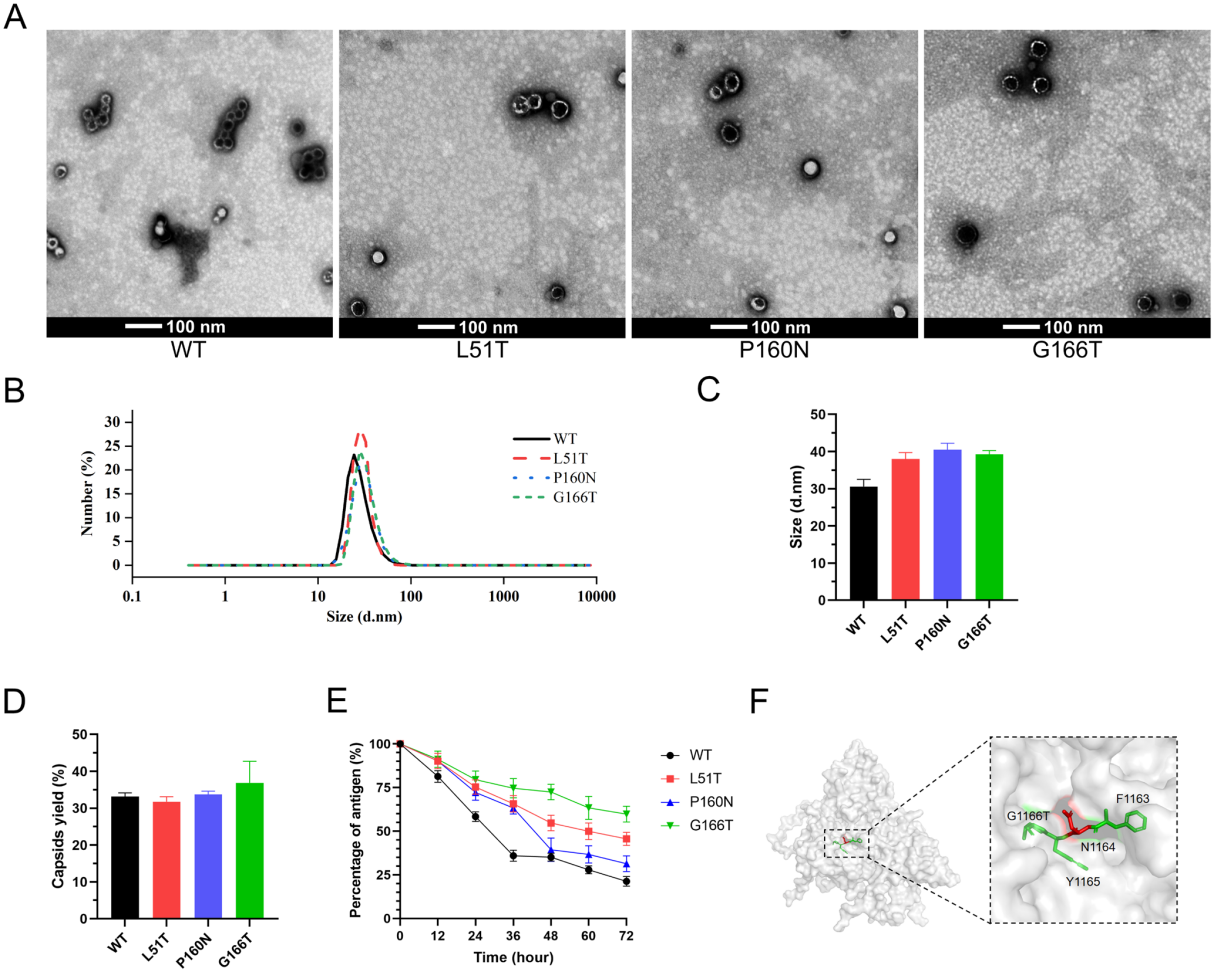
Assembly and Characterisation of WT VLPs and glycosylated VLPs. (A) TEM observation of VLPs. Scale bar shows 100 nm. (B) Analysis of VLPs by DLS. (C) Statistical data of the VLPs' size. The size was measured using Nano Measurer 1.2 and statistically analysed using GraphPad Prism 8. (D) Evaluation of VLPs assembly efficiency. (E) Analysis of VLPs stability by ELISA. VLPs with different amino acid changes in VP1 were incubated to assess stability and the intact VLPs were quantified by ELISA. (F) The location of G166T amino acid change formed the N‐glycosylation motif ‘FNYT’ within the 5S.

To confirm whether the secreted proteins can be assembled into VLPs, N‐glycosylated VP1, secreted VP3, and VP0 were assembled in vitro and then subjected to TEM observation and DLS assay. TEM shows spherical‐like particles, with slight shape irregularities compared to the ideal FMDV VLPs (Figure 3A). The assembly efficiency of WT VLPs was approximately 32%, while the glycosylated VLPs ranged from 30% to 35%. Notably, G166T VLPs showed slightly enhanced assembly efficiency compared to other mutants (Figure 3D). The results of the DLS assay (Figure 3B) revealed that the diameter of WT VLPs was 30 nm, consistent with previously reported (Acharya et al. 1989). Notably, the N‐glycosylated VLPs reached an average diameter of 35–40 nm (Figure 3C), indicating a size expansion likely attributed to the external display of N‐glycans (Lavado‐García et al. 2022). These findings illustrated that glycosylated and nonglycosylated VP1 can co‐assemble with VP3 and VP0 to form VLPs.

To verify the stability of glycosylated VLPs, samples were incubated at 25°C, and VLPs were quantified by ELISA at 12 h intervals. The results showed a minimal difference in the stability of L51T VLPs compared with that of WT VLPs. The time required for 50% dissociation of P160N VLPs was prolonged by more than 12 h. On the contrary, G166T VLPs exhibited the greatest stability, which was maintained for 72 h at 25°C, and the intact antigen remained at approximately 60% (Figure 3E). PyMOL analysis revealed that the N‐glycosylation motif ‘FNYT’ was made after the G166T amino acid change; this may be associated with the improved stability of VLPs (Figure 3F). These findings suggested that the N‐glycans on the surface of VLPs had a negligible effect on assembly efficiency but enhanced VLP stability.

### 3.4 N‐Glycan Profiling of VP1

**FIGURE 4:**
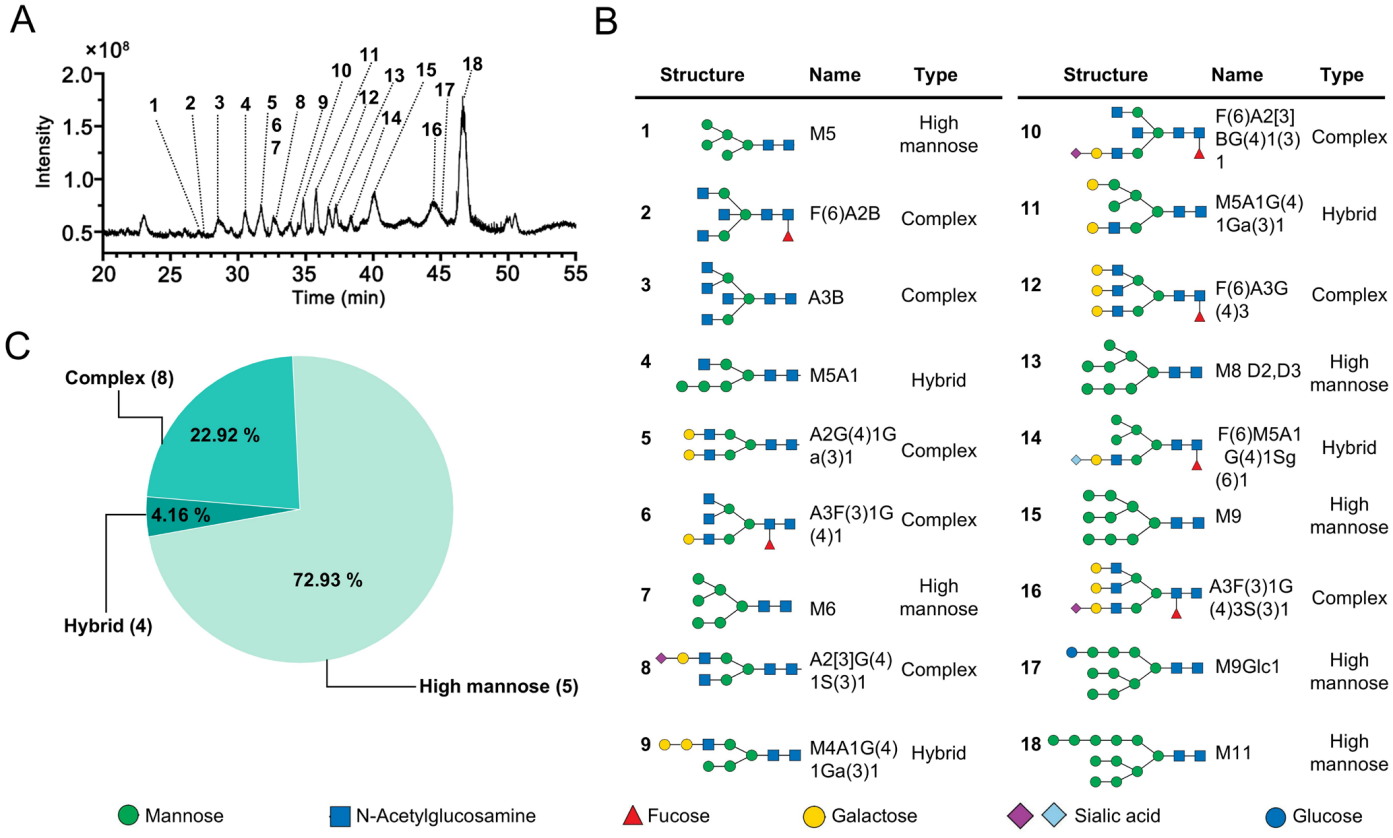
N‐glycan species analysis of the G166T VP1. (A) Ion chromatogram of N‐glycans released from G166T after PNGase F treatment. The ion peaks of the 18 glycans identified are labeled. (B) Structure and types of the 18 N‐glycans identified. A, Branch on the core structure of tri‐mannose; F, Fucose; G, Galactose (Gal); Glc, Glucose; M, Mannose (Man); S, Sialic acid. (C) Classification of these 18 N‐glycans identified in G166T VP1.

Glycosylated VP1 was analysed by LC–MS/MS analysis after digestion with PNGase F to analyse the N‐glycan species displayed on the surface of VLPs. A total of 18 N‐glycan species were identified and quantified from G166T VP1 (Figure 4A). Among these, eight were complex type, constituting 22.92%. The GlcNAc residues present on the antennae of these glycan structures could be sialylated or remain unsubstituted. The GlcNAc residue present in the core structure of the glycan could be fucosylated via α 1–3/6. Meanwhile, six of the 18 identified species belonged to the high‐mannose type, representing the greatest relative abundance (72.93%). These high‐mannose glycans were M5, M6, M8, M9, M11 and M9Glc1 glycans with isomers at termini. Both α (1–3) and α (1–6) arms contained only mannose residues in these structures. Furthermore, four hybrid glycans were identified, accounting for 4.16%. The α (1–6) arm of mannose at the end of the core structure of this group was identified, as was the α (1–3) arm, presented with modifications including GlcNAc, Gal and NeuAc (Figure 4B,C). The most abundant glycan identified was M_9_Glc_1_, with a relative abundance of 41.12%. This glycan was observed at m/z 1133.444 with a molecular weight of 2264.95 Da (Table S5).

### 3.5 Analysis of WT and Glycosylated VLP Uptake by DCs

**FIGURE 5:**
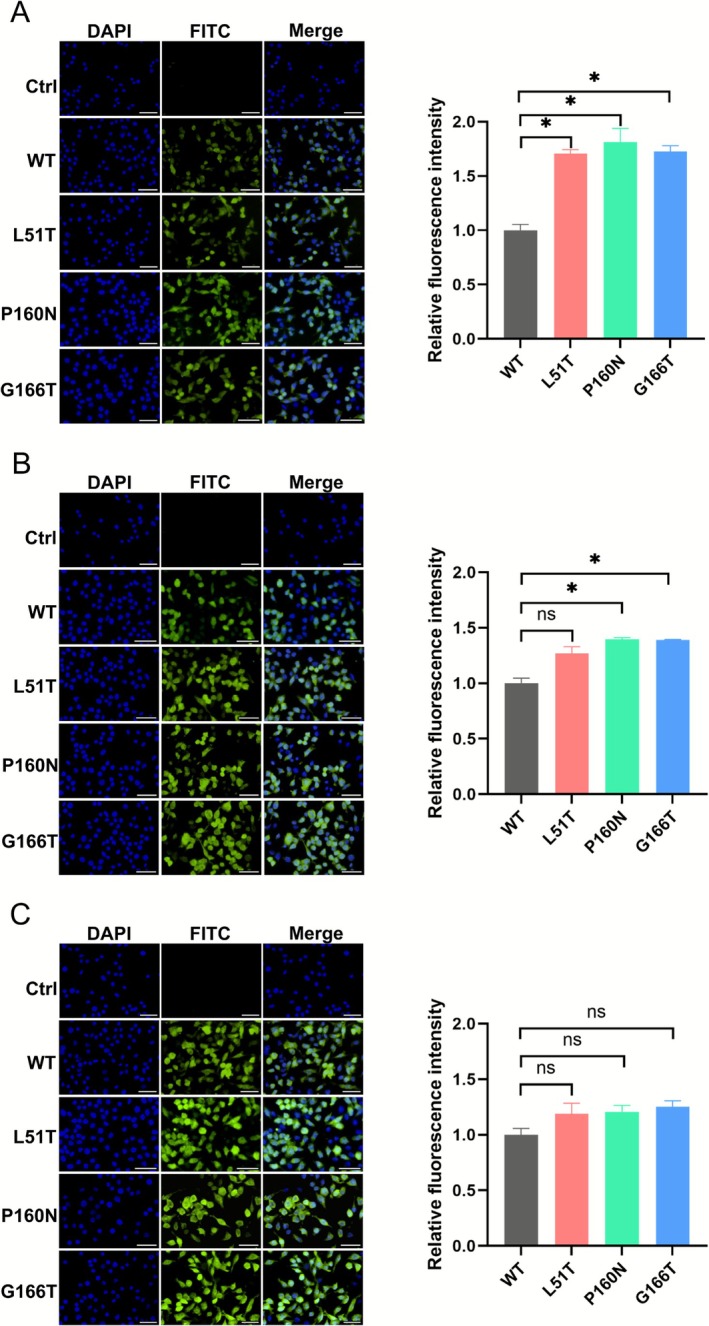
Uptake of VLPs by DCs. WT VLPs, L51T VLPs, P160N VLPs, and G166T VLPs were incubated in DCs for 1 h (A), 2 h (B), and 6 h (C), after which IFA was conducted using anti‐FMDV polyclonal antibody and FITC‐labelled goat anti‐pig IgG as the secondary antibody. The cell nuclei were stained with 4′,6‐diamidino‐2‐phenylindole (DAPI), and the DAPI and FITC fluorescence images were integrated into a merged image. The control group was incubated with PBS, the WT group was incubated with WT VLPs, the L51T group was incubated with L51T VLPs, the P160N group was incubated with P160N VLPs, and the G166T group was incubated with G166T VLPs, 1 bar = 100 μm. Representative data shown are mean ± SD from one of three independent experiments (*p < 0.05).

To verify the uptake of glycosylated VLPs by DCs, DCs were incubated with 1 μg of WT or glycosylated VLPs for 1, 2 or 6 h, respectively. After incubation, IFA was performed. The results demonstrated that the fluorescence intensity of the glycosylated VLPs was markedly greater than that of WT VLPs after 1 h incubation (Figure 5A) which was likely due to the mannose receptor (MR) of DCs facilitating the uptake process. After incubation for 2 h, the situation was similar. However, the fluorescence intensity of P160N was most pronounced (Figure 5B). Following a 6 h incubation, the fluorescence intensity in each group exhibited minimal variation, indicating that the uptake of VLPs was approaching saturation (Figure 5C). These findings suggested that glycosylated VLPs could be effectively recognised and internalised by DCs, and the fluorescence intensity in each group showed little significant difference at 6 h.

### 3.6 Assessment of the Immune Response of Glycosylated VLPs in Mice

**FIGURE 6:**
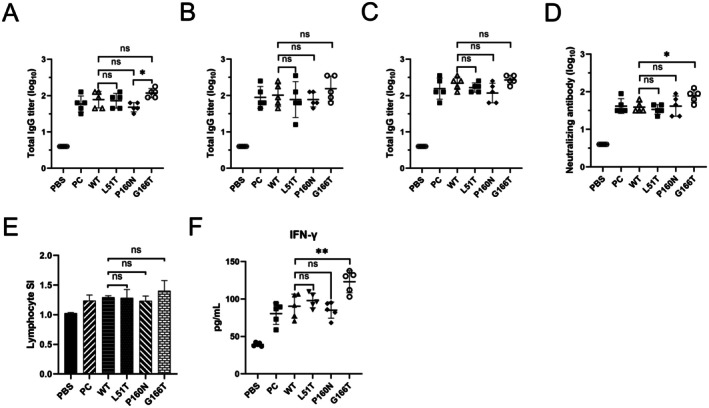
Evaluation of the immune response of glycosylated VLPs in mice. Blood samples were collected at 14, 28 and 42 dpi for antibody detection. Specific antibody levels were quantified by sandwich ELISA at 14 dpi (A), 28 dpi (B) and 42 dpi (C). (D) Neutralising antibody levels in serum of mice at 28 dpi (measured by microplate neutralising assay). (E) Detection of lymphocyte proliferation levels in mice at 28 dpi. (F) Detection of IFN‐γ levels at 28 dpi using ELISA kits. The statistical significance of differences was evaluated (*p < 0.05, **p < 0.01). Groups nomenclature are as follows: PBS: Negative control; PC, Positive control; L51T: L51T VLPs; P160N: P160N VLPs; G166T: G166T VLPs.

To test whether N‐glycans could influence the immunogenicity of VLPs, WT and glycosylated VLPs were emulsified with equal volumes of ISA 201 and administered via intramuscular injection. Blood samples were collected at 14, 28 and 42 dpi. The ELISA results demonstrated that the specific antibodies against FMDV in all experimental groups could be detected at 14 dpi, with titers exceeding 1:32 (Figure 6A), and high levels of antibodies could be maintained until 42 dpi. By contrast, no specific IgG was detected in PBS‐treated mice. In comparison with the positive control (PC) group, the yeast‐derived WT VLPs and glycosylated VLPs exhibited no significant difference. The effect of glycosylated VLPs on immunogenicity differed, with P160N eliciting a lower antibody response than the other two groups, in comparison with the effect of WT VLPs (Figure 6B,C). The neutralising antibodies against FMDV could be discerned after 28 dpi in the serum, exhibiting a pattern analogous to specific antibodies and showing a positive correlation. (Figure 6D).

The cellular immune response after VLP immunisation was evaluated using the lymphocyte proliferation assay and measurement of cytokine release. Three mice were randomly selected from each group for analysis via lymphocyte proliferation assay at 28 dpi. The results demonstrated that T‐cell responses were elevated in all experimental groups, but no significant difference was observed between WT and glycosylated VLPs compared with PBS (Figure 6E). The capacity of VLPs to induce cytokine production was evaluated by quantifying the serum IFN‐γ levels of mice at 28 dpi. Compared with the PBS controls, both WT and glycosylated VLPs resulted in significantly elevated levels of IFN‐γ after immunisation. In particular, G166T VLPs elicited the most pronounced stimulation of cytokine production (*p* < 0.01) (Figure 6F). These findings implied that glycosylated VLPs elicited not only enhanced humoral immunity but also augmented cellular immunity relative to WT VLPs.

### 3.7 Evaluation of the Immune Effect of Glycosylated VLPs in Pigs

**FIGURE 7:**
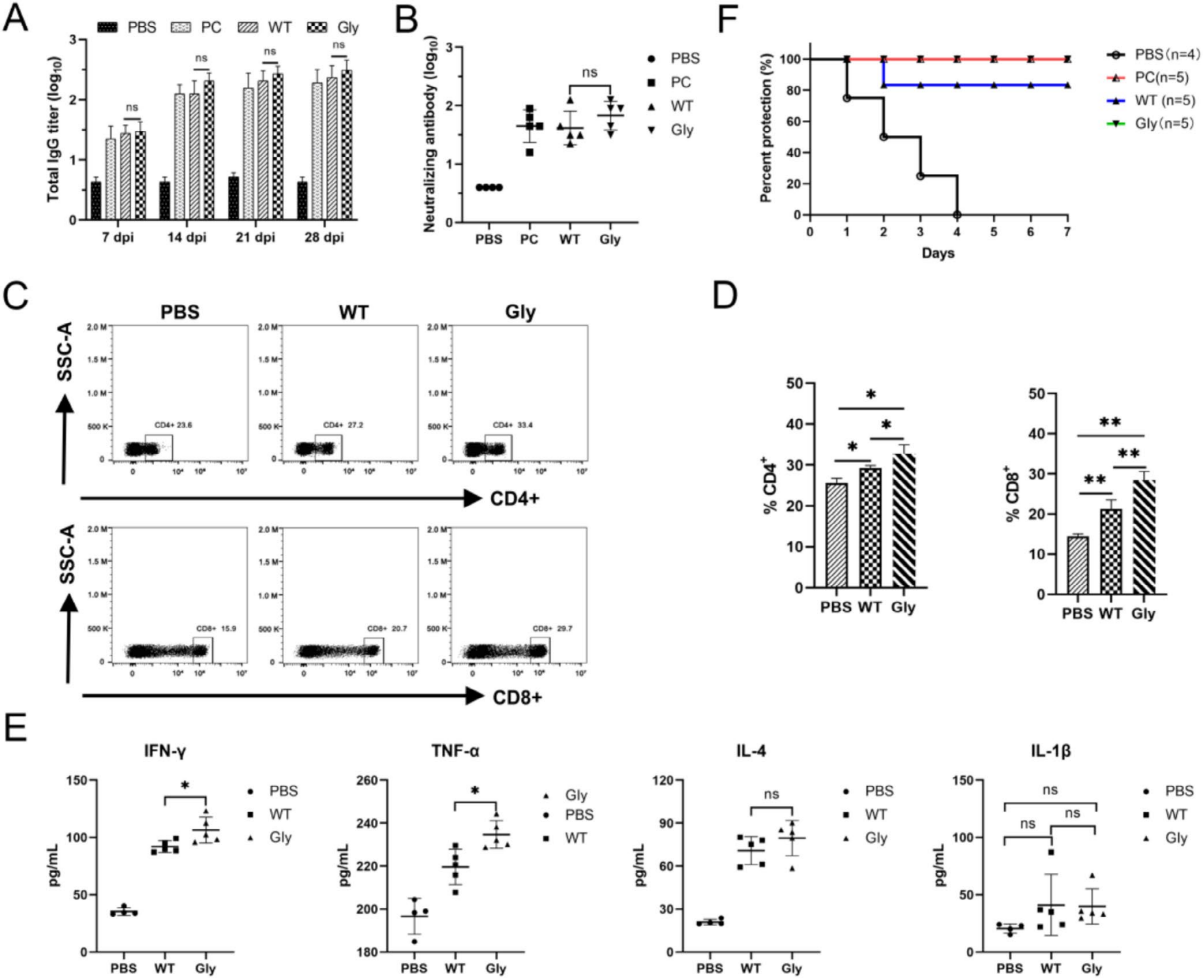
Evaluation of the immune effect of glycosylated VLPs in pigs. (A) The level of specific antibodies against FMDV was detected by sandwich ELISA in pigs. (B) The level of neutralising antibodies was detected by microplate neutralising assay in pigs at 28 dpi. PBMCs were isolated at 28 dpi, and CD4+ and CD8+ T cells in PBMCs were detected using flow cytometry (C and D). (E) The levels of IFN‐γ, TNF‐α, IL‐4 and IL‐1β induced by VLPs in serum samples from pigs at 28 dpi were detected using ELISA kits. The error bars indicate the standard deviation. Significant differences were calculated (*p < 0.05, **p < 0.01, ***p < 0.001). (F) Four weeks after immunisation, pigs were challenged and the protection was monitored.

To assess the immunogenicity of VLPs in pigs, *E. coli* ‐derived VLPs were used as a positive control, PBS as a negative control and sera were collected weekly to detect specific antibodies by sandwich ELISA. In addition, neutralising antibodies and cytokine levels were detected at 28 dpi. Specific antibody levels were detected on 7 days in WT and Gly (G166T VLPs) groups and increased gradually with time until 28 dpi. The mean specific antibody levels in the Gly group were not significantly different from the WT group (Figure 7A). The data revealed that the WT and Gly groups exhibited neutralising antibody titers exceeding 1:22 at 28 dpi, a level that was significantly higher than that observed in the PBS group. The mean neutralising antibody titre in the Gly group was higher than in the WT group (Figure 7B). To evaluate the lymphocyte activation in pigs by VLPs and glycosylation, PBMCs were isolated from immunised pigs at 28 dpi and analysed by flow cytometry. Cytokine levels (IFN‐γ, IL‐4, TNF‐α, IL‐1β) were also detected using ELISA kits. The results showed a notable alteration in the number of CD3^+^ CD4^+^ T‐lymphocytes among the immunised groups (Figure 7C). The percentage of CD4^+^ T‐lymphocytes exhibited a marked increase in the WT and Gly groups in relation to the PBS group. Twenty‐eight days following immunisation, the percentage of CD4^+^ T lymphocytes in the Gly group was 3.48% higher than the WT group (Figure 7D). Similarly, the percentage of CD8^+^ T lymphocytes was 7.28% higher in the Gly group than the WT group. The results of the cytokine assay revealed that the levels of IL‐4 were markedly elevated in the WT and Gly groups compared with those in the PBS group. No statistically significant difference was observed in IL‐1β levels, while the IFN‐γ and TNF‐α levels exhibited a slight increase in the Gly group in comparison with the WT group (Figure 7E). These findings indicated that yeast‐derived VLPs could elicit Th1‐ and Th2‐type immune responses in pigs. Moreover, glycosylated VLPs could elicit stronger cellular immunity than WT VLPs.

To ascertain whether VLPs confer protection against FMDV infection in pigs, the immunised pigs were challenged. All four animals in the PBS group exhibited overt clinical signs on the hoof and snout, whereas none of the five animals in the Gly group manifested any clinical signs and exhibited complete protection. However, one pig in the WT group exhibited a neutralising antibody titre at 1:22, yet displayed signs of haemorrhage lameness on the front hoof. Consequently, it was classified as unprotected, resulting in an overall protection rate of 80% (Figure 7F). These results indicated that both WT and glycosylated VLPs could stimulate high levels of protective antibodies.

## 4 Discussion

Currently, FMD still causes huge losses to the local livestock industry and economic development in some developing countries, especially low‐income countries (Maradei et al. 2013). Inactivated vaccines are the most effective vaccines for the prevention of FMD. However, their manufacturing process involves handling live viruses, which brings about concerns regarding safety and potential risks of virus leakage. On the contrary, VLPs are considered a highly safe and effective alternative but they still have certain concerns in the manufacturing process while VLPs are very safe and effective (Wang et al. 2016). To enhance or modulate the immunogenicity of the FMDV VLPs, biochemically modified VLPs by N‐glycosylation were produced. DCs, as highly effective APCs that can express substantial quantities of C‐type lectins, which have a high affinity for mannose, are capable of stimulating and sensitizing T cells to initiate an early immune response. Thus, VLPs glycosylation may influence the interactions with lectins, and consequently affect the uptake of VLPs, processing and presentation (Wolfert and Boons 2013).

The uptake of VLPs by APCs was enhanced by the mannose modification of rabbit hemorrhagic disease virus (Al‐Barwani et al. 2014). The hepatitis B virus (HBV) surface protein HBsAgS was modified with high mannose, leading to enhanced immunogenicity of VLPs (Joe et al. 2020). Thus, appropriate glycosylation of VLPs is an attractive approach for developing highly efficient and immunogenic vaccines.

Based on the previous studies, numerous signal peptides have been identified as suitable for efficient expression in *P. pastoris*, including α‐factor (Donelan et al. 2023), GAS, PHO5, FLO10 (Huang et al. 2011), DSE4 (Liang et al. 2013) and SP27 (Zou et al. 2022). To screen the signal peptides suitable for expressing structural proteins of FMDV, VP0 was selected as the model protein due to its highest expression level among the three structural proteins (Li et al. 2025). Signal peptide screening revealed that VP0 could be effectively secreted by the SP27 signal peptide (Figure 1D). As is known, proteins can enter the endoplasmic reticulum and Golgi apparatus for glycosylation. Despite containing three glycosylation sites, VP0 is not glycosylated, possibly because all sites are buried in its interior. Similarly, VP1 possessed a glycosylation site but was located within the α‐helix region, which may reduce susceptibility to glycosylation. This finding is consistent with previous studies that N‐glycosylation typically occurs in the loop or β‐turn position (Chen et al. 2016). Interestingly, when the structural proteins of enterovirus 71 were produced in insect cells, N‐glycosylation was observed at position 176 of VP1 (Zhao et al. 2017). As the primary antigenic protein of FMDV, VP1 contains numerous antigenic epitopes. Thus, we focused on exploring how VP1 glycosylation affects the immunogenicity of VLPs.

In this study, we identified 16 potential glycosylation sites by searching for the N‐glycosylation potential motif of VP1. Based on whether the site was external or in the loop region, three sites (L51T, P160N, G166T) were selected for mutation. This selection process was guided by previous research on protein glycosylation and structural biology, aiming to find sites that are more likely to undergo successful glycosylation. After induction and SDS‐PAGE analysis, the mutated VP1 exhibited a band migration of approximately 3 kDa. Subsequently, the band was reverted to its original size through the treatment of PNGase F, confirming the N‐glycosylation of mutated VP1. Subsequently, the G166T amino acid change was selected, and the LC–MS/MS analysis revealed that 72.93% of the N‐glycan species were high‐mannose type, consistent with previous reports (Bretthauer and Castellino 1999). The most abundant N‐glycan was M_9_Glc_1_, with a relative molecular mass of 2264.95 Da (Table S5), accounting for the ~3 kDa mobility shift observed in mutated VP1 (Figure 2C). CD analysis was performed to assess whether the secondary structure of VP1 was influenced by the glycosylation. The results revealed that the secondary structure of the mutants and WT exhibited minimal alteration.

To facilitate the purification of the VLPs, a His_6_ tag was inserted at residue 136 of VP1. This was based on the observation that the G‐H loop region of VP1 tolerates tag insertion without disrupting VLP assembly (Zhu, Yang, et al. 2018). The assembled VLPs displayed His_6_ tags on their surface, simplifying the complex sucrose density gradient purification (Porta et al. 2013). Glycosylated VP1, VP3, and VP0 were then assembled, and ELISA quantification showed no significant difference in the assembly efficiency between glycosylated and WT VLPs. We found that glycosylated VLPs have a slightly larger diameter than WT VLPs. As previously reported, *E. coli* ‐derived VLPs form 25–30 nm particles following in vitro assembly (Guo et al. 2013; Xiao et al. 2016). Thus, the increased VLP diameter is most likely attributed to N‐glycan display on the VLP surface. Notably, no cleavage of VP0 into VP2 and VP4 was observed in this study. VP0 cleavage depends on the active site formed by the conserved histidine (H2145) and whether the conformation of the adjacent sequence meets the characteristics of autocatalysis. The lack of VP0 processing may be related to the expression environment of VP0, which likely disrupted catalytic site formation, or the immediate analysis of VLPs after preparation that provided insufficient time for VP0 autocatalysis. Intact VP0 does not affect the assembly of FMDV VLPs, but this unprocessed state may be one of the reasons for the irregular morphology of the VLPs. VP0 cleavage can strengthen inter‐pentamer interactions and its absence may lead to structural heterogeneity (Curry et al. 1997). However, we have not compared these complexes of yeast‐derived VP0, VP3, and non‐glycosylated VP1 assembled in vitro. This would help identify the size differences between glycosylated VLPs and WT VLPs that might be caused by the expression system and in vitro assembly mode. We nevertheless plan to perform this comparison in the future. VLPs are assembled by noncovalent bonds, including hydrophobic interactions, electrostatic forces, and hydrogen bonding (Twomey et al. 1995). The instability of FMDV VLPs was mainly due to the protonation of amino acids near the 2‐fold and 3‐fold axis (Caridi et al. 2015). However, none of these amino acid changes were located near the interfaces. Notably, glycosylated VLPs exhibited enhanced stability, maintaining integrity for at least 3 days at 25°C. Glycosylation is hypothesized to have two main effects. First, the glycans may create a spatial hindrance to prevent protein aggregation and stabilize the protein (Vagenende et al. 2009). Second, the glycosylation may increase the hydrophilicity of the protein, enabling it to form hydrogen bonds with water and thus form a hydrated layer around the VLPs (Timasheff 1993). This may be related to the fact that FMDV VLP is stable in sugar solution (Yang et al. 2017). This study investigated the effect of VP1 glycosylation on VLPs and explored the potential glycosylation sites of VP0 and VP3. Future studies will aim to identify additional glycosylation sites near 2‐fold or 3‐fold axes that may modulate assembly, and explore methods for secreting the structural proteins or VLPs into the supernatant.

Uptake experiments were performed to detect the recognition and uptake of glycosylated VLPs by DCs in vitro. The uptake of glycosylated VLPs by DCs was enhanced over the first 6 h. This finding provided evidence that mannose‐modified VLPs can be rapidly taken up by DCs via the MR. This phenomenon was also demonstrated through in vivo experiments, in which glycosylated VLPs stimulated higher levels of specific antibodies than WT VLPs, indicating that glycosylation could enhance the immunogenicity of VLPs. The same trend was evident in the lymphocyte proliferation assay and IFN‐γ detection (Figure 6E,F).

Notably, P160N VLPs showed reduced antibody titers and lymphocyte SI, likely because the 140–160 aa region of the VP1 GH loop harbors a neutralizing epitope (Lee et al. 2018). The glycans at this site may impair the exposure of antigenic epitopes and cellular recognition (Tong et al. 2023). The G166T VLPs elicited a robust immune response in pigs, with 100% protection after challenge. Detection of neutralizing antibodies showed that all VLP‐immunized groups could reach high levels. Remarkably, the scatter plot of neutralizing antibodies showed that the maximum log_10_‐transformed antibody titre was 2.1, corresponding to an actual titre of 1:128 (Figure 7B). The results of the flow cytometry assay indicated that both Th1 and Th2‐type immune responses occurred in pigs immunized with VLPs. The glycosylation induced the proliferation of CD8^+^ T cells, which favored a cellular immune response. Cytokines were detected at 28 dpi, and the results showed significantly elevated IFN‐γ and TNF‐α in the Gly group compared with those in the PBS group. The mean levels of the Gly group were higher than the WT group. The insignificant difference in IL‐1β implied that no inflammatory storm of cytokines occurred in pigs immunized with Gly. The results demonstrated the importance of glycosylation modification in enhancing the immunogenicity of VLPs, which is characterized by a robust Th1 response after vaccination.

Overall, our study showed that appropriate glycosylation modification enhanced the stability and immunogenicity of VLPs. The significance of glycosylation in enhancing the characteristics of proteins and augmenting immunogenicity has been recognized (Sun et al. 2018). However, a considerable gap remains in exploring glycosylation in nonenveloped viruses. This study provides a method for developing superior vaccines by modifying the glycosylation sequon, enabling the precise design of polysaccharide‐modified vaccines that exert adjuvant synergistic effects. This approach offers a potential avenue for producing more advanced bioengineered vaccines.

## Author Contributions

**Zhiyao Li:** methodology, Data curation, Investigation, Formal analysis, Writing – original draft, Writing – review and editing, Visualization, Project administration; **Hu Dong:** methodology, Formal analysis, Resources, Supervision; **Shuanghui Yin:** methodology, Investigation, Resources; **Manyuan Bai:** methodology, Data curation, Validation; **Zhidong Teng:** methodology, Resources; **Lingbo Chen:** methodology, Data curation, Software; **Suyu Mu:** methodology, Investigation; **Yun Zhang:** data curation, Resources; **Yaozhong Ding:** resources, Supervision, Investigation; **Shiqi Sun:** supervision, Conceptualization, Resources; **Huichen Guo:** conceptualization, Supervision, Funding acquisition, Resources, Validation, Writing – review and editing.

## Funding

This work was supported by the Key R&D Program of Ningxia province (2024BBF02017). The Major Science and Technology Project of Gansu Province (24ZDWA004). Technology innovation guidance program of Gansu Province (24XNA030). Lanzhou Talent Innovation and Entrepreneurship Project (2023‐RC‐3). Local Financial Funds of National Agricultural Science and Technology Center, Chengdu (No. NASC2024KR06).

## Ethics Statement

All the animal experiments were performed with the approval and authorization of the Animal Welfare and Ethics Committee of Lanzhou Veterinary Research Institute, Chinese Academy of Agricultural Sciences (permit number RDDM‐0042‐00 and LVRI/HL/JL048‐02‐00), Lanzhou, China.

## Conflicts of Interest

The authors declare no conflicts of interest.

## Supporting information


**Figure S1:** Screening of potential glycosylation sites of VP1. (A) The secondary structure of VP1. The images were generated using the online software Novopro. Potential glycosylation sites are marked with red triangles. (B) The locations of the L51T, P160N, and G166T amino acid changes within the tertiary structure. The images were generated via PyMOL. Mutant sites were marked red; VP0 is magenta, VP1 is green, and VP3 is yellow.
**Figure S2:** CD analysis of VP1 and its mutants.
**Table S1:** Screen potential glycosylation sites of VP1 through “N”.
**Table S2:** Screen potential glycosylation sites of VP1 through “T”.
**Table S3:** Screen potential glycosylation sites of VP1 through “S”.
**Table S4:** Secondary structure of VP1 and the mutants.
**Table S5:** N‐glycan Information of G166T.

## Data Availability

The data that support the findings of this study are available from the corresponding author upon reasonable request.
