# Supplementary material for: Glycosylated Foot‐And‐Mouth Disease Virus‐Like Particles Produced in Pichia Pastoris Enhance Stability and Immunogenicity
Source: Microb Biotechnol. 2025 Nov 24;18(11):e70271. doi: 10.1111/1751-7915.70271 (PMC12642819; doi:10.1111/1751-7915.70271)
Supplement: Supplementary file 1 — Figure S1: Screening of potential glycosylation sites of VP1. (A) The secondary structure of VP1. The images were generated using the online software Novopro. Potential glycosylation sites are marked with red triangles. (B) The locations of the L51T, P160N, and G166T amino acid changes within the tertiary structure. The images were generated via PyMOL. Mutant sites were marked red; VP0 is magenta, VP1 is green, and VP3 is yellow. Figure S2: CD analysis of VP1 and its mutants. Table S1: Screen potential glycosylation sites of VP1 through “N”. Table S2: Screen potential glycosylation sites of VP1 through “T”. Table S3: Screen potential glycosylation sites of VP1 through “S”. Table S4: Secondary structure of VP1 and the mutants. Table S5: N‐glycan Information of G166T. [file MBT2-18-e70271-s001.docx]

**Supplementary**

Fig. S1. Screening of potential glycosylation sites of VP1. A. The secondary structure of VP1. The images were generated using the online software Novopro. Potential glycosylation sites are marked with red triangles. B. The locations of the L51T, P160N, and G166T amino acid changes within the tertiary structure. The images were generated via PyMOL. Mutant sites were marked red; VP0 is magenta, VP1 is green, and VP3 is yellow.


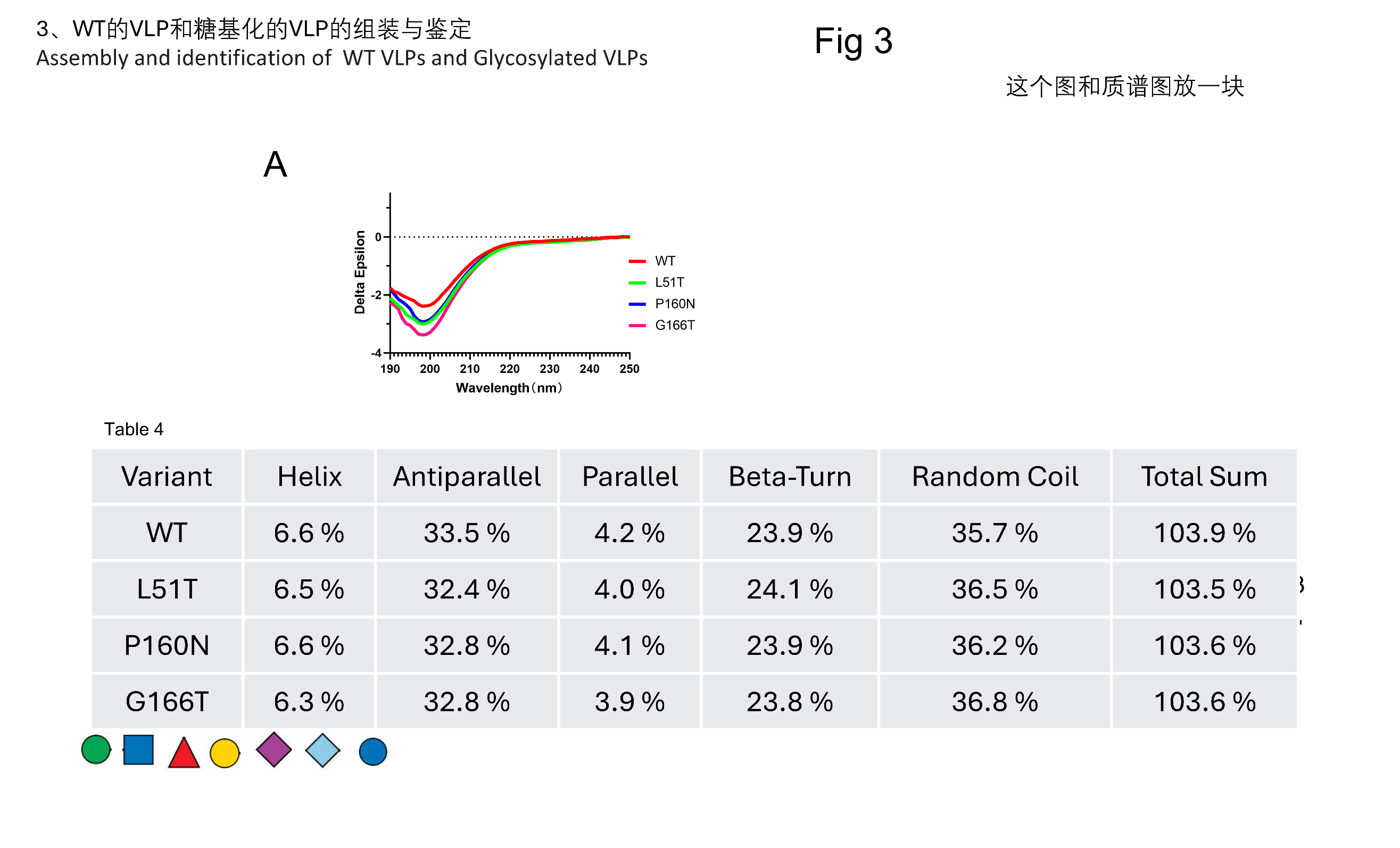


Fig. S2. CD analysis of VP1 and its mutants.Table S1. Screen potential glycosylation sites of VP1 through "N".

| Index | Mutation | Mutation Energy (kcal/mol) | Effect |
| --- | --- | --- | --- |
| 1 | GLY166>THR | -0.14 | NEUTRAL |
| 2 | GLY19>THR | -0.03 | NEUTRAL |
| 3 | LEU51>THR | 0.03 | NEUTRAL |

Table S2. Screen potential glycosylation sites of VP1 through "T".

| Index | Mutation | Mutation Energy (kcal/mol) | Effect |
| --- | --- | --- | --- |
| 1 | ASP85>ASN | -0.69 | STABILIZING |
| 2 | THR2>ASN | -0.55 | STABILIZING |
| 3 | THR12>ASN | -0.14 | NEUTRAL |
| 4 | ALA183>ASN | -0.12 | NEUTRAL |
| 5 | HIS28>ASN | -0.08 | NEUTRAL |
| 6 | PRO10>ASN | 0.28 | NEUTRAL |
| 7 | PRO118>ASN | 0.34 | NEUTRAL |

Table S3. Screen potential glycosylation sites of VP1 through "S".

| Index | Mutation | Mutation Energy (kcal/mol) | Effect |
| --- | --- | --- | --- |
| 1 | GLY5>ASN | -0.70 | STABILIZING |
| 2 | ASP31>ASN | -0.66 | STABILIZING |
| 3 | THR1>ASN | -0.24 | NEUTRAL |
| 4 | THR56>ASN | 0.01 | NEUTRAL |
| 5 | PRO160>ASN | 0.03 | NEUTRAL |
| 6 | HIS195>ASN | 0.07 | NEUTRAL |

Table S4. Secondary structure of VP1 and the mutants.

| Variant | Helix | Antiparallel | Parallel | Beta-Turn | Random Coil | Total Sum |
| --- | --- | --- | --- | --- | --- | --- |
| WT | 6.6% | 33.5% | 4.2% | 23.9% | 35.7% | 103.9% |
| L51T | 6.5% | 32.4% | 4.0% | 24.1% | 36.5% | 103.5% |
| P160N | 6.6% | 32.8% | 4.1% | 23.9% | 36.2% | 103.6% |
| G166T | 6.3% | 32.8% | 3.9% | 23.8% | 36.8% | 103.6% |

Table S5. N-glycan Information of G166T.

| Type | No. | RT | M/Z | Charge St. | Mono Mass Exp. | Avg Mass Exp. | MS Area |
| --- | --- | --- | --- | --- | --- | --- | --- |
| M5 | 2 | 28.38 | 727.809 | 2 | 1453.604 | 1454.26 | 8.64E+05 |
| F (6)A2B | 3 | 28.75 | 943.404 | 2 | 1884.7941 | 1885.56 | 1.33E+05 |
| A3B | 4 | 29.52 | 971.915 | 2 | 1941.8157 | 1942.79 | 1.04E+06 |
| M5A1 | 5 | 30.28 | 829.349 | 2 | 1656.6826 | 1657.39 | 2.68E+05 |
| A2G (4)1Ga (3)1 | 6 | 31.45 | 930.889 | 2 | 1859.7628 | 1860.7 | 1.36E+06 |
| A3F (3)1G (4)1 | 7 | 31.68 | 1024.431 | 2 | 2046.8483 | 2047.87 | 2.03E+06 |
| M6 | 8 | 31.72 | 808.835 | 2 | 1615.6561 | 1616.35 | 2.41E+06 |
| A2[3]G(4)1S (3)1 | 9 | 32.35 | 995.912 | 2 | 1988.8068 | 1989.81 | 1.25E+05 |
| M4A1G (4)1Ga (3)1 | 10 | 33.22 | 910.376 | 2 | 1818.7367 | 1819.63 | 1.96E+05 |
| F (6)A2[3]BG (4)1S (3)1 | 11 | 34.46 | 780.655 | 3 | 2337.9399 | 2339.05 | 7.31E+05 |
| M5A1G (4)1Ga (3)1 | 12 | 35.33 | 991.403 | 2 | 1980.7905 | 1981.77 | 2.85E+05 |
| F (6)A3G (4)3 | 13 | 36.13 | 791.659 | 3 | 2370.9517 | 2372.05 | 1.88E+05 |
| M8 D2, D3 | 14 | 36.87 | 970.889 | 2 | 1939.7627 | 1940.73 | 4.38E+05 |
| F (6)M5A1G (4)1Sg (6)1 | 15 | 38.01 | 758.636 | 3 | 2271.8831 | 2273 | 3.02E+05 |
| M9 | 16 | 39.26 | 1051.915 | 2 | 2101.8162 | 2102.84 | 3.47E+05 |
| A3F (3)1G (4)3S (3)1 | 17 | 43.93 | 1332.532 | 2 | 2662.0491 | 2663.26 | 1.88E+05 |
| M9Glc1 | 18 | 44.52 | 1133.444 | 2 | 2263.8704 | 2264.95 | 1.04E+07 |
| M11 | 19 | 46.46 | 1214.47 | 2 | 2425.9236 | 2427.06 | 3.12E+06 |
